# Root causes of COVID-19 data backlogs: a mixed methods analysis in four African countries

**DOI:** 10.1093/oodh/oqae009

**Published:** 2024-05-06

**Authors:** Emily Carnahan, Austin Van Grack, Brian Kangethe, Mamadou Mballo Diallo, Dominic Mutai, Oury Bah, Hassan Mtenga, Constant Kingongo, Julia Ngomba, Jessica Shearer, Joy Kamunyori, Robert Rosenbaum, Colleen Oakes, Maya Rivera Hildebrand, Matthew Morio, Mira Emmanuel-Fabula

**Affiliations:** Center of Digital and Data Excellence, PATH, 2201 Westlake Ave Suite 200 Seattle, WA 98121, USA; Research Department, Maternal & Infant Health Consulting, 1449 E Winder Lane, Salt Lake City, Utah, 84124-1401; Center of Digital and Data Excellence, PATH, Megacity Plaza, Mezzanine Floor Along Nairobi-Kisumu Road, Kisumu 40123, Kenya; Center of Digital and Data Excellence, PATH, Africa Outsourcing 131 Cité Keur Gorgui, 6eme étage immeuble Auchan 99999, Dakar, Senegal; Noncommunicable Diseases, PATH, A.C.S. Plaza, 4th floor Lenana Road and Galana Road, Nairobi 00100, Kenya; Center of Digital and Data Excellence, PATH, Africa Outsourcing 131 Cité Keur Gorgui, 6eme étage immeuble Auchan 99999, Dakar, Senegal; Center of Digital and Data Excellence, PATH, Oasis Office Park, 3rd Floor Off Haile Selassie Rd, Oyster Bay, Dar es Salaam, Tanzania. PO Box 13600; DRC Country Program, PATH, Matrix Tower Building 119 Blvd du 30 Juin, 5th Floor, Gombe, Kinshasa Kinshasa Congo, Democratic Republic of the Congo; DRC Country Program, PATH, Matrix Tower Building 119 Blvd du 30 Juin, 5th Floor, Gombe, Kinshasa Kinshasa Congo, Democratic Republic of the Congo; Health Systems Innovation and Delivery, PATH, 455 Massachusetts Ave NW, Suite 1000 Washington, DC 20001, USA; Bureau for Global Health, United States Agency for International Development, GHTASC-Credence, 500 D Street SW, Washington, DC, 20024, USA; Bureau for Global Health, United States Agency for International Development, GHTASC-Credence, 500 D Street SW, Washington, DC, 20024, USA; Bureau for Global Health, United States Agency for International Development, GHTASC-Credence, 500 D Street SW, Washington, DC, 20024, USA; Center of Digital and Data Excellence, PATH, Rue de Varembe 7 Foundation for Appropriate Technology in Health, 1202 Geneva, Switzerland; Medical Devices and Health Technologies, PATH, 2201 Westlake Ave Suite 200 Seattle, WA 98121, USA; Market Dynamics, PATH, Rue de Varembe 7 Foundation for Appropriate Technology in Health, 1202 Geneva, Switzerland

**Keywords:** digital health, COVID-19, COVID-19 vaccination data, Africa, root cause analysis, data backlog

## Abstract

With the unprecedented scale and scope of the COVID-19 vaccination response, many countries used digital systems to capture vaccine administration data. Data backlogs, a build-up of information captured via paper forms not yet entered into digital systems, were common across countries. This study aimed to identify the root causes of COVID-19 vaccination data backlogs in the Democratic Republic of the Congo, Kenya, Senegal and Tanzania based on primary (interviews and observations at vaccine delivery sites) and secondary data. Root causes of data backlogs were related to technology (system slowdowns, insufficient devices and limited system functionality), infrastructure (lack of reliable internet and data bundles), processes (incongruence between paper and digital tools, separate data collection and entry, lack of integration with routine immunization and lack of standard operating procedures) and people (staff shortages, large workloads and non-payment of staff). Recommendations to inform digital and data systems include: (i) use a country-led, coordinated, iterative approach for system design and introduction, (ii) start with a minimum viable product and (iii) proactively address the needs of the health workforce. As the COVID-19 global emergency ends, these findings can help inform broader health system strengthening efforts to improve effectiveness, resilience and pandemic preparedness.

Abrégé

Face à l’ampleur et à la portée sans précédent de la campagne vaccinale contre la COVID-19, de nombreux pays ont utilisé des systèmes numériques pour recueillir des données sur l’administration du vaccin. Tous les pays ont connu des retards de données, soit une accumulation d’informations recueillies au moyen de formulaires papier qui n’avaient pas encore été saisies dans les systèmes numériques. Cette étude vise à cerner les causes premières de ces retards de données sur la vaccination contre la COVID-19 en République démocratique du Congo, au Kenya, au Sénégal et en Tanzanie sur la base de données primaires (entretiens et observations sur les sites d’administration du vaccin) et secondaires. Les causes premières des retards de données étaient liées à la technologie (ralentissements des systèmes, insuffisance des appareils et fonctionnalité limitée des systèmes), à l’infrastructure (absence de forfaits de données et de liaison Internet fiables), aux processus (incompatibilité entre les outils papier et numériques, collecte et saisie séparées des données, manque d’intégration avec les vaccinations de routine et absence de procédures opérationnelles normalisées) et au personnel (manque d’effectifs, charges de travail importantes et non-paiement du personnel). Les recommandations pour informer les systèmes numériques et de données comprennent: (i) utiliser une approche itérative, coordonnée et dirigée par les pays pour la conception et la mise en place des systèmes, (ii) commencer par un produit minimum viable et (iii) répondre aux besoins du personnel de santé en amont des problèmes. La crise mondiale liée à la COVID-19 touchant à sa fin, ces résultats sont susceptibles d’éclairer les efforts de renforcement des systèmes de santé dans leur ensemble visant à améliorer l’efficacité, la résilience et la préparation aux pandémies.

Resumen

Ante la escala y el alcance sin precedentes de la vacunación como respuesta a la COVID-19, muchos países utilizaron sistemas digitales para registrar datos sobre la administración de las vacunas. En todos los países era común que la información recabada a través de formularios en papel se acumulara sin ingresarse en los sistemas digitales. El objetivo de este estudio consistió en identificar las causas fundamentales de la acumulación de datos sin procesar sobre la vacunación contra la COVID-19 en la República Democrática del Congo, Kenia, Senegal y Tanzania a partir de datos primarios (entrevistas y observaciones en los sitios de vacunación) y secundarios. Las causas fundamentales de esa acumulación estaban relacionadas con la tecnología (desaceleraciones del sistema, dispositivos insuficientes y funcionalidad limitada del sistema), la infraestructura (falta de conexión confiable a Internet y paquetes de datos), los procesos (incongruencia entre las herramientas en papel y las digitales, recopilación e ingreso de datos por separado, falta de integración con la inmunización de rutina y falta de procedimientos operativos estándar) y las personas (escasez de personal, grandes cargas de trabajo y falta de pago del personal). Las siguientes son algunas recomendaciones para los sistemas digitales y de datos: (i) utilizar un enfoque iterativo, coordinado y dirigido por el país para el diseño y la introducción del sistema, (ii) comenzar con un producto mínimo viable y (iii) atender de manera proactiva las necesidades del personal de la salud. Ahora que está terminando la emergencia mundial de COVID-19, estos hallazgos pueden servir de base a esfuerzos más amplios de fortalecimiento del sistema de salud dirigidos a mejorar la efectividad, la resiliencia y la preparación para pandemias.

## INTRODUCTION

Routine health management information systems (HMISs) are an essential health system component for capturing information and informing decision-making to drive health system performance [1, 2].

Integrating digital health technologies and/or the data collected into these systems has the potential to improve service delivery by reducing manual record-keeping and labor, improving data quality, making data more accessible for decision-makers and providing clinical decision support [3–9]. To achieve their intended impact, digital health technologies must be appropriate for the local context and supported by an enabling environment [10, 11].

With the onset of the COVID-19 pandemic, countries had to quickly determine how to best capture and use COVID-19 data, whether through new, adapted or expanded HMISs. The availability of COVID-19 vaccines launched the most rapid global vaccine rollout in history [12]. To aid this rollout, many countries attempted to capture digitized data at the individual level and on a large scale—some for the first time and rapidly. Digitized individual-level, longitudinal data can support tracking patients over time (e.g. for a multidose vaccine series) and across service delivery sites. These data also enable new ways of assessing program performance with more granular data that can be disaggregated in various ways (e.g. by time, patient characteristic, administrative unit or vaccine dose) [13, 14].

A 2021 report found that electronic immunization registries (digital systems that capture individual-level, longitudinal data on vaccine doses administered) had been piloted or implemented in more than 50 low- and middle-income countries [14]. Many of these systems were introduced prior to the pandemic and focused on routine childhood immunizations, which constituted a different target population and service delivery model than COVID-19 vaccination.

Since COVID-19 vaccines first became publicly available in late 2020, digital systems have been integral to many countries’ COVID-19 vaccine implementation efforts. Digital systems have been used to collect patient-level or aggregate data on vaccine administration, vaccine availability, adverse events following immunization and cases of vaccine-preventable diseases [15, 16]. They also have been used to provide individuals with certificates for proof of vaccination [15–18].

However, due in part to the rapid nature of the COVID-19 vaccine rollout and the newness of the digital system being deployed, many countries experienced significant data backlogs—a build-up of paper-based information not yet entered into the intended digital system used to capture information about COVID-19 vaccination administration. High-quality data are essential to inform vaccination planning and service delivery [19, 20]. Data backlogs undermine the availability of complete and timely data, impacting frontline workers’ ability to follow up with individuals due for subsequent doses in a series and impacting health managers’ ability to accurately assess vaccination coverage rates, target supportive supervision visits or forecast stock needs. Incomplete data may contribute to a lack of trust in the data, limiting its use and thereby undermining the effectiveness and efficiency of the health system.

Data backlogs and related digital and data challenges are not specific to the COVID-19 pandemic or vaccination response. The 2014–17 West Africa Ebola epidemic was characterized by suboptimal data quality, management and sharing that delayed the epidemic response [21, 22]. Underlying challenges included weak infrastructure (electricity and/or internet), lack of foundational datasets (e.g. unique identifiers), limited digitization of data, non-aligned data standards and a lack of interoperability between data systems [21]. Despite these challenges, information and data use were essential to the epidemic response [21].

In the same way that lessons from the Ebola epidemic have informed health system strengthening efforts and the COVID-19 pandemic response [21, 22], lessons from the COVID-19 pandemic can be used to improve effectiveness and resilience of country health systems and prepare for future pandemics.

Understanding and addressing data backlogs is crucial for any large-scale data collection effort, including the collection of routine individual patient-level data (e.g. for HIV, malaria, or immunization), aggregate data for health sector monitoring and short-term and/or high-volume data related to an emergency response or campaign (e.g. immunization campaigns and contact tracing). In any context, stopgap solutions can address current backlogs, but to enable digital systems to function optimally, it is necessary to identify and address the root causes that create the backlogs. If these drivers are not addressed, we will continue to sink resources into stopgap solutions or the data may never be digitized for use.

In the context of COVID-19 vaccination, in May 2022, the United States Agency for International Development (USAID) conducted a rapid internal data collection exercise to identify challenges with patient-level vaccine administration data across 42 countries receiving USAID funding for COVID-19 vaccines through the US Government’s Initiative for Global Vaccine Access. Initial analysis found that technology (software offline functionality and hardware access), infrastructure (internet and electricity), processes (data management) and people (workforce and training) were primary drivers of COVID-19 data backlogs.

Building on USAID’s preliminary analysis, this study aimed to identify the root causes of COVID-19 vaccination data backlogs in the Democratic Republic of the Congo (DRC), Kenya, Senegal and Tanzania and recommend actions to address those causes. Specific research questions were as follows:

(1) What were the root causes that contributed to COVID-19 vaccine administration data backlogs and how did the root causes differ by context?
(2) What can we learn from COVID-19 vaccine administration data backlog root causes that can inform the design of digital and data systems and processes across routine and other immunization platforms?

## METHODS

A mixed methods study was conducted in DRC, Kenya, Senegal and Tanzania in partnership with each country’s ministry of health (MOH). Digital Square led the study in Kenya, Senegal and Tanzania, and the MOMENTUM Routine Immunization Transformation and Equity project led the study in DRC; both projects are funded by USAID.

This study was part of the COVID-19 Vaccine Digital Collaborative Learning Agenda convened by USAID. The study team participated in regular meetings of the Learning Agenda consortium; the methods and results were used to inform the Learning Agenda theory of change and responses to the Learning Agenda’s questions [23].

### Setting

DRC, Kenya, Senegal and Tanzania were purposefully selected for this study because they were the countries where USAID funded Digital Square and the MOMENTUM Routine Immunization Transformation and Equity project to strengthen COVID-19 vaccine digital and data systems. Each country’s COVID-19 vaccine data systems are described further below.

### DRC

DRC recorded its first COVID-19 case in March 2020; as of 12 July 2023, it had recorded 97 697 cases and 1468 deaths [24], although these figures are likely underreported [25]. Since receiving the first shipment of COVID-19 vaccines in March 2021, DRC initially focused on vaccinating priority populations but then shifted to the general population by the time of this study. COVID-19 vaccination services have been available at health facilities and clinics across much of DRC since April 2021. Additionally, both mobile COVID-19 vaccination campaigns and mass vaccination campaigns have been implemented [26].

DRC's government assessed various options for recording, managing and reporting COVID-19 vaccination data and opted to deploy the District Health Information Software 2 (DHIS2) Tracker. DHIS2 Tracker was the tool recommended by the COVID-19 Vaccines Global Access (COVAX) initiative and the World Health Organization [27, 28]. The module enables entry of individual patient data and can send second-dose reminders, produce patient ‘due’ lists and produce digital vaccine certificates. Tracker was somewhat familiar to stakeholders in DRC (from use in other health areas and COVID-19 surveillance) and is interoperable with the national DHIS2; however, the decision to adopt Tracker was based on the perception that DRC needed to achieve certain functional requirements (e.g. client registration, appointment reminders, vaccine certificates and analysis of individual-level data), which were ultimately not used [29]. Hardware, infrastructure and human resource challenges resulted in suboptimal use of Tracker; in April 2021, the Ministry of Public Health, Hygiene and Prevention developed and implemented a parallel Microsoft Excel-based reporting database, which required manual aggregation of data at each level of the health system, resulting in incomplete data. DHIS2 Tracker and Excel were both in use for COVID-19 vaccine administration reporting at the time of this study.

### Kenya

Kenya recorded its COVID-19 index case on 12 March 2020; by 12 July 2023, there were 343 786 confirmed cases and 5689 deaths [24]. With COVID-19 vaccines becoming available globally at the beginning of 2021, the government rolled out the National COVID-19 Vaccines Deployment and Vaccination Plan to guide vaccination deployment, implementation and monitoring countrywide [30].

For routine immunization data, most service delivery sites in Kenya use a paper-based approach where healthcare workers (HCWs) capture patient-level data on paper-based registers and aggregate the number of vaccine doses delivered on a tally sheet. Tally sheets are used to complete a monthly summary report. After vaccination, each patient is issued a vaccination card showing vaccination details, including the next due date. In some subnational areas, electronic immunization registries for routine immunization data have been implemented using digital systems such as OpenSRP and OpenMRS [14].

The Kenya MOH and Ministry of Information, Communication and Technology jointly developed Chanjo Kenya (ChanjoKE), an online-only custom-built electronic immunization registry as a monitoring system for COVID-19 vaccination data to mimic routine immunization data flow processes [31]. ChanjoKE provides health facilities with analytics for reporting and supply chain management and allows clients to directly access the online platform to register themselves, schedule vaccinations and obtain vaccination certificates.

In February 2021, initial training on ChanjoKE was rolled out nationwide, targeting national, county and subcounty Expanded Programme on Immunization coordinators. Subsequent 3-day training sessions were conducted by county and subcounty health management teams targeting HCWs from major vaccinating health facilities. During the same month, the National Vaccines and Immunization Program distributed tablets and SIM cards as needed to hospitals, health centers and dispensaries countrywide, enabling the formal use of ChanjoKE for COVID-19 vaccination reporting.

Reporting rates showed the use of ChanjoKE in major hospitals but inconsistent use in smaller hospitals, health centers and dispensaries. The intent was for HCWs to enter data directly into ChanjoKE and not use paper registers for COVID-19 vaccine administration data. However, in practice, HCWs adopted a dual data entry system in which they created their own paper-based tools to capture data and used personal mobile devices for ChanjoKE data entry.

### Senegal

Senegal recorded its first case of COVID-19 on 2 March 2020 [25]; as of 12 July 2023, it had recorded 89 007 cases and 1971 deaths [24]. The country implemented measures to deal with the pandemic, from instituting a curfew to closing markets and borders. COVID-19 vaccination began on 23 February 2021, first targeting high-priority groups and subsequently extending to everyone over 18 years of age. According to the country’s COVID-19 vaccination training guide, since January 2022, a booster dose has been offered to anyone who had completed their vaccination series at least 6 months prior.

Senegal uses DHIS2 as the national HMIS and began using Tracker for COVID-19 case management and contact tracing at the onset of the pandemic based on the recommendation of the Health Information System Division [32]. Once COVID-19 vaccines became available, the Ministry of Health and Social Action adapted and implemented the existing paper-based routine immunization data collection tools for COVID-19 vaccine administration data. Using the same data flow process as routine immunization, health facility staff captured COVID-19 patient-level data on paper registers and then aggregated and entered the data into DHIS2. The Ministry of Health and Social Action chose to digitize aggregate (and not individual-level) data to quickly deploy a familiar reporting process to capture the initial influx of COVID-19 data. This followed the same process used for routine immunization data, except that COVID-19 vaccination data were reported daily and routine immunization data were reported monthly. For this reason, the paper registers and DHIS2 modules for inputting COVID-19 vaccine data and routine immunization data were separate. The DHIS2 aggregate system required internet connectivity and did not support offline data entry.

### Tanzania

Tanzania recorded its first COVID-19 case on 16 March 2020, in Arusha [33]; as of 12 July 2023, it had reported 43 078 cases and 846 deaths [24]. The government activated the Epidemic Response Team to implement interventions to curb the spread of COVID-19 through case detection, contact tracing, testing, case management, infection prevention and other public health measures [33]. COVID-19 vaccination began in July 2021 and nationwide vaccination campaigns were initiated in September 2021 [34]. During this time, health facilities used the existing manual routine vaccination system (patient cards, registers, tally sheets and monthly summary reports) to record patient-level data as a stopgap measure for managing COVID-19 data, while the government developed alternative data management platforms. Staff in some regions had previous experience using the Tanzania Immunization Registry, an electronic immunization registry for routine immunizations that was first introduced in 2016 [9].

In February 2022, the Ministry of Health, Community Development, Gender, Elderly and Children launched the revised National COVID-19 Response Plan, aligned with the National Immunization Strategy (2020–25). Based on the plan, and in collaboration with non-governmental organizations, the government implemented a web-based electronic system for vaccine data management, Chanjo COVID [35], a DHIS2 Tracker-based platform for management of vaccination appointments, vaccination administration and certificate issuance and verification. At its launch, Chanjo COVID was implemented nationwide at the district council level and at major health facilities, including hospitals and vaccinating health centers. Some provinces piloted the COVID-19 module integrated into the OpenIZ-based Tanzania Immunization Registry (TImR). The government provided tablets and data bundles to enable data entry into Chanjo COVID, as the system required internet connectivity for data entry. (Since this study concluded, the Ministry of Health, Community Development, Gender, Elderly and Children has added offline functionality to Chanjo COVID.) Health facilities that could not complete data entry (e.g. due to issues with devices, internet access, system functionality and staff bandwidth) delivered their paper forms to council health management teams (CHMTs) for entry into Chanjo COVID. Regional health management teams (RHMTs) provided technical support and oversight for data management.

### Study design

The study used primary and secondary data to identify the root causes of data backlogs in each of the four countries. First, study teams in each country analyzed secondary data including vaccine stock and aggregate vaccine administration data, then conducted primary data collection through observations and interviews at health facilities. In parallel, study teams conducted national-level technical assessments. The study teams used root cause analysis to synthesize findings and identify the root causes of data backlogs [36].

### Sampling

Within each country, subnational administrative areas that received COVID-19 vaccines for distribution were purposefully selected in collaboration with each country’s MOH.

In Kenya, Senegal and Tanzania, four to six regions/counties were selected to include a mix of urban and rural settings and a range of the size (number of records) of data backlog (based on secondary data). Within each region, four to six health facilities were selected to maximize variations in urban–rural settings, facility types, facility ownership, catchment population, infrastructure (different levels with regard to electricity and internet availability) and vaccine data backlog (facilities with existing/growing backlog versus those with cleared or significantly lower backlog). Within each facility, one or two individuals were sampled for interviews based on their roles in COVID-19 vaccine service provision (e.g. HCW) or data management (e.g. data entry clerk). Table 1 summarizes the sampling approach for health facilities and respondents.

**Table 1:** Facility and respondent sampling framework for each region in Kenya, Senegal and Tanzania

**Facility**	**Facility characteristics**	**Facility interview respondents (#)**
Facility #1, urban, with backlog	Required characteristics: • High-volume facility with large catchment area • Identified as having an existing and/or growing data backlog Preferred characteristics: • Additional staffing to support data entry • Relatively stable electricity and internet connectivity	Health worker (1) Data entry clerk (1)
Facility #2, urban, no backlog	Required characteristics: • High-volume facility with large catchment area • Identified as having cleared its data backlog and/or has a significantly lower backlog than comparable facilities Preferred characteristics: • Additional staffing to support data entry • Relatively stable electricity and internet connectivity	Health worker (1) Data entry clerk (1)
Facility #3, periurban, with backlog	Required characteristics: • Medium-volume facility with relatively stable electricity and internet connectivity • Does not have staff to support data entry; data entry is completed by HCWs • Identified as having an existing and/or growing data backlog	Health worker (2)
Facility #4, periurban, no backlog	Required characteristics: • Medium-volume facility with relatively stable electricity and internet connectivity • Does not have staff to support data entry; data entry is completed by HCWs • Identified as having cleared its data backlog and/or has a significantly lower backlog than comparable facilities	Health worker (2)
Facility #5, dispensary	Required characteristics: • Low-volume facility that delivers vaccines Preferred characteristics: • Relatively unstable electricity and limited internet connectivity and/or captures immunization records on paper	Health worker (1)
Facility #6, rural/other	Required characteristics: • Low-volume facility that delivers vaccines Preferred characteristics: • Relatively unstable electricity and limited internet connectivity requiring paper capture of immunization records	Health worker (1)

**Table 2:** Data collection samples by country

**Country**	**Region/County/Province**	**Number of facilities/respondents**	**Facility setting**	**Facility type: % (N)**	**Data collection timeline**
Democratic Republic of the Congo	Province: Kinshasa	6 health facilities/7 respondents	Urban: 100%	Hospitals: 50% (3) Dispensaries and clinics: 17% (1) Primary health care facilities: 33% (2)	August 2022 (review of data from April to June 2021)
Kenya	Counties: Busia, Homabay, Kajiado, Kakamega, Kilifi and Laikipia	50 health facilities/50 respondents	Urban: 42% Semi-urban: 6% Rural: 52%	Dispensaries and clinics: 38% (19) Hospitals (primary and secondary): 30% (15) Primary health care facilities: 24% (12) Comprehensive teaching and referral hospitals: 8% (4)	October–December 2022
Senegal	Region: Kédougou	6 health facilities/6 respondents	Urban: 50% Rural: 50%	General high-volume health care faciliies: 100% (6)	November 2022
Tanzania	Regions: Dar es Salaam, Dodoma, Rukwa and Songwe	67 health facilities/80 respondents	Urban: 49% Rural: 51%	Hospitals: 21% (14) Health centers: 33% (22) Dispensaries: 46% (31)	September 2022

In DRC, the study team selected 2 of 35 health zones in the province of Kinshasa based on data and geographic accessibility, leadership support for the study and collaboration with the MOMENTUM Routine Immunization Transformation and Equity project. One health zone was high performing and the other low performing according to data record completeness (with only urban sites included as the study was implemented in an urban province).

### Data collection

Data collection was conducted by local study teams, ranging from one to six individuals in each country. Secondary data on COVID-19 vaccine stock and administration were provided by the MOH. Primary data collection occurred at health facilities that were service delivery points for COVID-19 vaccinations. In each facility, study teams observed patient and data workflows if COVID-19 vaccinations were taking place and conducted interviews with facility staff.

In Kenya, Senegal and Tanzania a semi-structured data collection tool was developed as a guide for primary data collection at health facilities. This tool was further adapted to each country’s context. The data collection tool included an observation guide to capture real-time information on the status of devices, tools, infrastructure, systems, workflows and processes involved in COVID-19 vaccine service delivery and data collection. The tool also included a semi-structured interview guide to gain insight into respondents’ understanding of the broader system and workflow, including human resources, training, capacity, data management and data demand and use. The same interview guide was used for interviews with individuals in service provision and data management roles.

Study teams in Kenya, Senegal and Tanzania also conducted national-level technical assessments focusing on system configuration and performance issues, which included interviews with MOH and information and communications technology (ICT) staff. In Tanzania, additional interviews were conducted with management staff from both CHMTs and RHMTs, given their role in supporting data management.

In DRC, the MOMENTUM project collaborated with the World Health Organization to adapt the Performance of Routine Information System Management assessment tools for this study [37]. The resulting tools captured similar constructs to those used in the other three countries but also included a structured data quality assessment tool to compare paper-based data with data in DHIS2 Tracker and the Excel database. The DRC team also drew on documentation from multiple national meetings on the topic and from national strategies and plans, including the Ministry of Public Health’s Plan to Improve COVID-19 Vaccination Data.

Primary data were collected in 2022 in DRC (August), Tanzania (September), Senegal (November) and Kenya (October through December). In DRC, the primary data collection conducted in August 2022 reviewed vaccination data from April through June 2021; these data were triangulated with contemporary evidence from document review and supportive supervision documentation.

Table 2 summarizes the data collected in each country. In Senegal, data collection was hampered by a nationwide HCW strike (i.e. withholding of data reporting while still providing clinical care); data collection was possible in only one region where not all HCWs were on strike.

### Data analysis

The study teams in each country triangulated secondary sources of quantitative data on vaccines distributed and vaccines administered to estimate the size of data backlogs (number of records) at national and subnational levels and reviewed the results with MOH officials.

Qualitative semistructured data collected through interviews and observations at subnational sites were analyzed using Dedoose. Data were first analyzed for country-specific findings. In Kenya, Senegal and Tanzania, a single analyst led content and thematic analyses of the qualitative data and quantified the occurrence of common themes. In DRC, pairs of individuals within the study team analyzed the data by theme before sharing findings with the full study team for confirmation and further contextual information, where available. A participatory root cause analysis (using Miro) was conducted with the study teams to combine cross-country findings and identify causal pathways and root causes [36].

The study team categorized root causes as related to technology, infrastructure, processes or people. ‘Technology’ refers to digital and data services and applications, their design and functionality and the equipment/hardware used to operate them. ‘Infrastructure’ refers to the networks and services that support electronic information exchange. ‘Processes’ refer to the action/steps to support the design or implementation of systems. ‘People’ refer to personnel, training, skills and capacity.

Recommendations to address the root causes were generated by the research team based on the study results and the team’s digital health expertise and knowledge of the local context. Recommendations were reviewed and validated by the MOHs in all countries and, in DRC, by other technical partners.

### Ethics

This study received non-human subjects research determination from PATH’s Research Determination Committee. Permission to conduct data collection at health facilities was sought from each country’s MOH. The data were stored to ensure the privacy and confidentiality of participants.

## RESULTS

**Figure 1:**
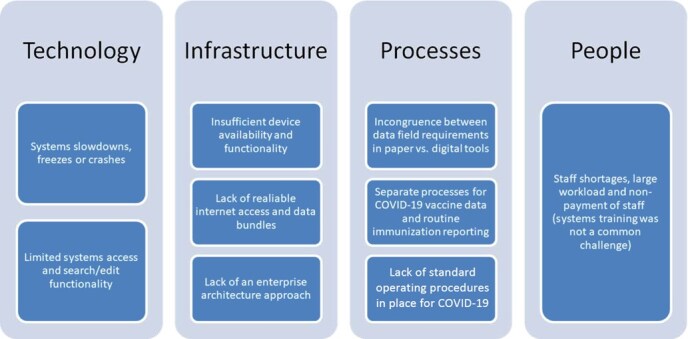
Summary of root causes of data backlogs by category

This study identified multiple, interrelated root causes of COVID-19 vaccine administration data backlogs that were common across countries (Fig. 1). This section presents the root causes organized by the following categories: technology, infrastructure, processes and people.

### Technology

#### System slowdowns, freezes or crashes

In Kenya, Senegal and Tanzania, slowdowns, freezes or crashes were common challenges across COVID-19 vaccine data management systems due to weak internet, poor-quality devices or server overload. Respondents in all three countries (and across regions and facility types) reported that the COVID-19 data reporting systems used were slow to load, often stopped functioning and required reboots or restarts to function. This affected how quickly and when data could be captured in the system, contributing to data backlog.

Specifically, in Kenya 28 of 48 respondents (58%) noted that ChanjoKE would ‘hang’ or slow down, impeding data entry. Slow or weak internet (an infrastructure root cause) was perceived to contribute to system crashes or slowdowns. During the observation sessions in Senegal, in 75% of facilities (four of six) users experienced system freezes, had to reboot the application and/or noted other bugs within the system. Additionally, facility staff cited device quality and age as reasons for system freezes and crashes. In Tanzania, out of 43 respondents, 24 (56%) reported the software crashed either every day or every time it was used, while 19 (44%) experienced the same at least once a week. In all three countries, server overload was identified as the primary cause for system slowdown due to too many users at once and/or limited server capacity.

System crashes were not an issue in DRC, where DHIS2 Tracker was functional in the sites visited; however, these were all urban sites with reliable internet. Ongoing support from the University of Oslo also contributed to Tracker functionality.

#### Limited system access and search/edit functionality

Facility users in Kenya and Tanzania reported that the COVID-19 vaccine digital systems did not include search functionality. They said that this prevented users from finding patients in the system via identifying information (e.g. name or phone number), which could result in duplicate entries for patients or require additional staff time to search records manually, slowing the data entry process and contributing to data backlogs. Some noted that this negatively influenced staff motivation to perform data entry due to frustration with working within the limits of the digital system.

Respondents in both countries also reported that there was no way for users to edit patient records once they had submitted the data. One respondent in Kenya stated, ‘Facilities were not able to update [records] in the Chanjo system initially due to access rights.’ This created challenges for users who wanted to update or correct data after initial data entry, which, respondents said could lead to duplicate records and other data quality issues. Respondents from many facilities commented that having editing permission and search functionality would improve data entry processes.

In Senegal, aggregate data from paper forms were entered into DHIS2, so respondents did not note the same challenges with patient-level search and edit functionalities. In DRC, interviewees noted issues with DHIS2 Tracker user access—many users did not have login access when their facility received a tablet to begin using Tracker.

### Infrastructure

#### Lack of reliable internet access

In all four countries, the digital systems for COVID-19 vaccination data required online data entry at the time of this study (in Tanzania, Chanjo COVID has since been updated to include an offline mode).

Findings from interviews and observations indicated that the primary infrastructure challenge across countries was the lack of available or reliable internet access, impeding timely data entry by requiring system users to wait until connectivity was restored or to travel to an area with better network coverage to enter data.

In Kenya, 58% of facility respondents (28/48) reported network slowdowns or connectivity issues as a reason for data backlogs. In Tanzania, half of CHMT/RHMT respondents (12/24) reported having internet access between 1 and 3 days a week; 42% (10/24) reported having daily internet access. During observations in Senegal, both urban and rural facilities had internet access during the sessions; however, one urban and one rural facility had three disruptions (of over one minute) that were attributed to connectivity.

#### Lack of data bundles

Accessing the online systems via mobile devices required data bundles (packages to purchase mobile data) for internet connectivity. Facility respondents in Kenya, Senegal and Tanzania said they lacked sufficient data bundles required for online data entry due to inadequate budget for data bundles and lack of timely procurement or distribution of data bundles.

In Kenya, respondents at 94% of facilities (45/48) mentioned lack of data bundles as a challenge contributing to slow data entry, as staff were unable to access the online data systems or ended up purchasing data bundles themselves. At least 47% of respondents in Tanzania (21/45), 50% in Senegal (3/6) and 100% in two districts in Kenya (Kilifi, 10/10; Homa Bay, 11/11) reported having to personally pay for internet or data bundles to enter data into the systems.

Lack of reliable internet access or data bundles was not raised as an issue by respondents in the urban study sites visited in DRC.

#### Insufficient device availability and functionality

Across countries, our study found that most devices used by health facility staff were personal ones. According to respondents, facility-owned devices, when provided, were often older and non-functional due to issues with computer viruses, limited device memory, outdated or corrupted SIM cards, dead batteries or faulty charging ports. This lack of consistent access to functional devices slowed data entry and the ability to enter real-time data, contributing to data backlogs.

In Kenya, the most cited problem with MOH-provided devices was SIM card issues. Many of these devices were tablets procured for the 2015 census which had outdated operating systems and outdated or corrupted SIM cards. One healthcare worker noted, ‘We conduct outreaches but use our own resources (devices and data bundles).’ In DRC, respondents described delays in sites’ receiving tablets for data collection due to delayed disbursement of Gavi COVID-19 vaccine delivery support. In Tanzania, respondents said that most facilities had access to shared devices for data collection (with an average of two people per device), but staff preferred to use personal devices to improve efficiency. Although the use of personal devices may improve the timeliness of data entry, it can place a financial burden on staff (i.e. paying for data bundles) and lead to data privacy issues.

### Processes

#### Incongruence between the design of paper tools and digital system design

In all four countries, staff first collected COVID-19 data on paper registers and then transferred data to digital systems. In DRC and Senegal, the data fields in the paper and digital registers were well aligned: in DRC, the DHIS2 Tracker was based on the paper tool and in Senegal, aggregate data entry in DHIS2 mirrored data entry for routine immunization.

This was not the case in Kenya and Tanzania where paper tools were not initially made available for COVID-19 vaccine data capture. In Tanzania, when COVID-19 vaccines were first introduced, most respondents reported adapting routine immunization paper registers to record COVID-19 vaccine data because COVID-19 vaccination registers were not available. Similarly, in Kenya, facility staff reported using routine immunization paper registers or blank paper to record COVID-19 vaccinations during large outreach and mass campaign events since no standard paper tool had been provided.

Both countries developed digital systems in parallel to HCWs adapting or improvising paper tools. As a result, the format, questions and information collected on the paper registers did not align directly with those in the digital systems. Inconsistencies between paper and digital registers contributed to data backlogs because respondents said they were unsure how to proceed when the digital registers required data fields that were not in the paper forms, delaying data entry and requiring staff to track down the missing information.

#### Separate processes for COVID-19 vaccine data collection and data entry

In Kenya, Senegal and Tanzania, as well as some sites in DRC, the study team found that data capture first happened on paper registers and then was later entered into the digital system. Respondents noted that this practice led to delays between paper-based data capture and digital data entry steps and that the delay was especially common in vaccination campaign settings where large volumes of vaccinations were delivered in a short period of time without sufficient staff for timely data entry. Respondents also noted that when different staff were responsible for data collection on paper and data entry in the digital system, there were delays if the person entering the data could not read the handwriting on the paper forms or the paper forms were missing essential data elements required for digital entry, necessitating follow-up with the data collector(s).

Furthermore, in some sites in DRC, Kenya and Tanzania, data collection and data entry staff were in different locations. Transferring paper forms from one location to another caused delayed data entry and data backlogs.

We have 14 immunizing facilities, and not all have data clerks, and so they bring their data here [to the subcounty hospital] in hard copy. Sometimes they do not bring the data to be entered to Chanjo on time. The facilities are very far; the facility staff have to plan how the data will be brought here for data entry. – Respondent in Kenya

This was also true in Tanzania, where respondents mentioned that transport of the paper registers to the data entry location was inconsistent or untimely, especially for campaigns.

#### Separate processes for COVID-19 and routine immunization reporting

All four countries introduced COVID-19 vaccination data systems that were separate from routine immunization reporting. In DRC, Tracker presented a new workflow that differed from routine immunization, which health workforce respondents shared was challenging. The introduction of a parallel Excel database further increased their workload. In Senegal, respondents shared that even though the aggregate data fields and form layout were similar between COVID-19 and routine vaccination reporting, the systems for inputting the data remained separate and they were expected to enter COVID-19 vaccination data daily (versus monthly for routine immunization), thereby adding to their workload. In Kenya and Tanzania, ChanjoKE and Chanjo COVID, respectively, were new digital systems, separate from the routine immunization systems. Respondents described that the new systems and workflows required end-user training and added to their reporting burden.

#### Lack of standard operating procedures in place for COVID-19

Facility respondents in all four countries reported that they did not have standard operating procedures (SOPs) for COVID-19 vaccine service delivery and data management. Study observations showed that inconsistent data collection and entry processes were being applied across facilities. Study observations also showed that lack of SOPs, especially in terms of transferring data from paper tools to the digital system, impacted how quickly data were entered, and the lack of streamlined processes added to the time spent on data collection and entry, contributing to data backlogs.

Additionally, respondents in Kenya and Tanzania shared that there were no system user manuals, so end users did not know how to troubleshoot issues that could prevent them from entering data. In DRC, a user manual was created but not widely disseminated.

Despite the lack of COVID-19-specific SOPs, most facility respondents in Senegal (6/6, or 100%) and Kenya (32/37, or 86.5%) reported that processes were not more difficult for COVID-19 than for routine immunization. In practice, most facility respondents in Kenya (25/36, or 69%) and Tanzania (37/67, or 55%) said they relied on existing SOPs and guidelines for routine immunization to inform COVID-19 vaccination activities.

### People

#### Staff shortages, large workload and non-payment of staff

In all four countries, respondents cited staff shortages, large staff workloads and non-payment of staff as factors that led to health facility staff burnout or low motivation to perform data management activities. As a result, they reported that data entry did not happen in real time and data backlogs were not prioritized for later entry without dedicated support (e.g. from temporary data clerks).

In Kenya, respondents from most facilities reported feeling overburdened with work and having limited staff capacity. They also said that staff who performed vaccinations usually were also responsible for data entry, despite their large service delivery workload. In some cases, they noted that there was insufficient time in the day to complete all the work, so entering data often got postponed, contributing to the data backlog. As one respondent described, ‘I sometimes get overwhelmed with data entry and sometimes get [a] backlog. Sometimes I try and stay up to midnight to do data entry.’ Other respondents felt they had sufficient support during outreach activities when additional staff were mobilized but they felt overburdened during regular workdays. Healthcare workers in Kenya shared that they saw their role as clinical, while data entry was an administrative role, leading them to prioritize service delivery over data entry.

In Senegal, the motivation for the ongoing HCW strikes was attributed to difficult working conditions and large workloads for facility HCWs given additional data entry tasks. As a result, HCWs blocked transmission of COVID-19 vaccine data from the health facility to the district level during the period of this study. Half of the facility respondents interviewed (3/6) reported low motivation to do their jobs.

In Tanzania, HCWs reported low motivation for data management activities, largely due to non-payment of staff or high workload. Some respondents commented that motivation for data collection was higher for outreach and campaigns compared to fixed post/routine vaccination because additional compensation was offered to HCWs during outreach efforts and campaigns. However, HCWs said they had not received additional compensation as promised, which undermined their motivation. Lack of motivation to perform data entry led to delay of data entry and data backlog. Respondents also reported a shortage of facility staff and staff being overwhelmed and overworked, contributing to burnout and limiting the time HCWs had for data entry. As one Tanzania respondent summarized, ‘The biggest reason [for data backlogs] is the lack of manpower; let’s say the shortage of workers at some facilities.’

In DRC, data clerks or vaccinators did not enter data consistently into DHIS2 Tracker, particularly in public sector sites (e.g. health facilities), which lacked the dedicated data entry clerks found in private sector sites. Based on document review and observation, campaign sites funded by external partners also typically had paid data clerks. A root cause of low motivation in data entry staff was not being paid their salaries—partly due to delays in disbursement of Gavi funding but also due to DRC’s entrenched system in which health workforce salaries are frequently paid late [29]. Another contributing factor was the lack of supportive supervision in study sites to encourage or provide accountability for data entry.

#### Sufficient systems training in most countries

Staff in Kenya, Senegal and Tanzania shared that they received sufficient training on their respective digital systems, they felt comfortable entering data into the systems and they did not consider systems training as a root cause of data backlogs. However, in DRC, the initial training on DHIS2 Tracker largely took place online and as such did not include a hands-on component, resulting in many staff not having sufficient skills to use Tracker.

In Kenya, 94% of respondents (45/48) indicated that the training provided was sufficient, although some noted there was a lot of information covered in a short period. On average, the training duration was 2.3 days (compared with the planned 3-day training). Despite feeling comfortable with the system, 25% of facility respondents (12/48) suggested that a refresher training might be helpful.

In Tanzania, 80% of facility respondents (54/67) and 88% of CHMT/RHMT respondents (21/24) had been trained on Chanjo COVID, with an average training duration of 1.8 days and at least 2 staff trained at each facility.

In Senegal, no respondent received training on DHIS2 for COVID-19, but all respondents said they felt comfortable with the system. On average, respondents had been using the DHIS2 system for over 4 years.

## DISCUSSION

### Root causes of COVID-19 data backlogs

In response to the first research question (What were the root causes that contributed to COVID-19 data backlogs and how did the root causes differ by context?), the study identified root causes related to technology, infrastructure, processes and people that contributed to observed COVID-19 data backlogs (Fig. 1). Most were common across the four study countries, despite different contexts and different digital systems in use. The root causes were similar regardless of whether the digital system captured aggregate vaccination data (as in Senegal) or individual-level data (as in the other three countries).

The root causes were interrelated and mutually reinforcing, within and across categories. System slowdowns or crashes (technology) were in part due to poor internet connectivity (infrastructure), and the absence of system user manuals or troubleshooting guides (processes) limited HCWs’ ability to adequately address such system issues. The system slowdowns (technology), along with limited system search/edit functionality (technology), also contributed to HCW frustration and decreased motivation to use the digital system (people). The lack of data bundles and insufficient device availability and functionality (infrastructure) meant that many HCWs purchased their own data bundles and used their personal devices for data entry; this was not only a financial burden but also contributed to HCWs feeling unsupported by the health system, which limited their motivation to perform data entry (people). Multiple root causes added to HCWs’ workload (people), including additional reporting burden due to the lack of streamlined processes for COVID-19 and routine immunization reporting (processes), time spent tracking down missing data due to incongruence between the paper and digital tools (processes) and time spent manually searching records due to limited system search functionality (technology).

This article is not the first to identify these challenges with health system data collection through HMISs. Data backlogs limit data timeliness—a critical aspect of data quality. A 2020 scoping review on factors that limit the quality of data in immunization programs in low- and middle-income countries identified challenges related to tools (HMIS structural weaknesses and unreliable internet access), governance (lack of data management policies and processes) and people (lack of HCW capacity, limited supervision and feedback and low motivation) [20]. Similarly, an evidence synthesis from the Strategic Advisory Group of Experts on Immunization found that ‘root causes associated with poor data quality include gaps in health worker capability and motivation, performance-based targets, unsupportive leadership, lacking a culture of data use, poor information system design, overly complex tools, inadequate policies and resources and suboptimal processes for data collection and reporting, including supervision and feedback’ [19]. A 2017 literature review emphasized local-level human resources—capacity shortages and skills as primary root causes of poor immunization data [38]. The fact that some of the root causes identified in this study are already known indicates the need to continue focusing resources on addressing these challenges to strengthen health systems and ongoing pandemic preparedness.

In contrast to these previous studies, we found that HCW skills and capacity to use the digital systems were not a common root cause of data backlogs. In three of the four countries, HCWs reported sufficient capacity to use the digital systems. In Senegal, respondents attributed this to previous experience with DHIS2, whereas in Kenya and Tanzania, respondents attributed this to sufficient training. (HCWs’ use of the digital systems was observed by the study team and self-reported through interviews, but their skills were not tested or validated.)

### Recommendations to inform digital and data systems and processes

In response to our second research question (What can we learn from COVID-19 vaccine administration data backlog root causes that can inform the design of digital and data systems and processes across routine and other immunization platforms?), the study team identified three overarching recommendations based on the study results. First, country leaders should collaborate with donors to use an intentional and coordinated approach to design and introduce digital and data systems with planned iteration over time. This iterative design process should start not only with an assessment of tools and systems that exist within the country, but also those tools and systems that may be viable and are interoperable with existent systems—creating an ecosystem of choice prior to tool/system selection. Donors and implementing partners should promote a shared understanding of operational challenges and incorporate flexibility into project design. Second, when introducing new systems or workflows, stakeholders should start with a minimum viable product (MVP) and design the system based on the existing enabling environment. Third, country leaders and development partners should proactively address the needs of the health workforce given their essential role as end users of digital and data systems. When implemented collectively, these recommendations can address many of the root causes observed in this study. These recommendations can also be applied to address or prevent data backlogs in other large-scale data collection efforts. Below, each recommendation is described further. 

Countrywide digital and data systems, like those for COVID-19 vaccine data management, should be designed and implemented through an intentional, coordinated approach led by the appropriate national coordinating body. The national coordinating body should establish clear governance and accountability and engage a range of stakeholders—including those with digital and data expertise—who can advise on system design and implementation. A collaborative approach to identify business processes, data flows and requirements should guide system development [10, 39]. Process root causes identified in this study highlight the importance of designing digital systems aligned to reporting requirements and data workflows. We note that a thorough collaborative approach is not always feasible in an emergency response when there is a tradeoff between rapidly introducing a system to meet urgent needs and taking time for upfront, intentional design. However, even after systems are introduced, our findings indicate that stakeholders should prioritize reviewing and iteratively updating service delivery processes, paper forms and digital systems to streamline data reporting, rationalize data elements and align paper and digital tools. This intentional and iterative approach could address many of the process root causes identified in this study. As part of this approach, the national coordinating body should identify who is responsible for maintaining and updating SOPs and guidance documents. Other studies have recommended the development of flexible national plans to guide vaccine program rollout that can be adapted in an emergency response context [40].

Given the need to rapidly introduce new systems or workflows in a pandemic setting, stakeholders should start with an MVP. An MVP is a version that has ‘just enough features to satisfy early users, meet the minimum functionalities, and to provide feedback for future releases of the product’ [41]. The critical data elements and functionalities to include in the MVP should be identified through the rapid, collaborative approach led by the appropriate national coordinating body as described earlier. Critical data elements should be prioritized based on their importance to inform decision-making. In an emergency response, donors or multilateral organizations should prioritize providing timely guidance to countries on the MVP requirements to support global reporting requirements or cross-country data sharing.

The MVP (and subsequent system iterations) should be designed based on the existing enabling environment, defined by the eHealth building blocks of leadership and governance; strategy and investment; legislation, policy and compliance; workforce; infrastructure; standards and interoperability and services and applications [42]. Importantly, many of the root causes identified in this study could have been addressed had the current country context been considered in the design and implementation of COVID-19 vaccination data systems and processes. For example, country leaders should assess the current infrastructure limitations; if there are challenges with internet connectivity, systems should have an offline option for data entry. An assessment of server capacity and device availability could address technology-related root causes. When introducing or scaling a digital system, country leaders should ensure sufficient server capacity to avoid system slowdowns, freezes or crashes. Each server should meet the minimum requirements for the system and load capacities, which may require the purchase of new or extended servers. Use of the system also requires sufficient functional devices (e.g. phones, tablets, or computers) and technical support for end users. Country leaders should consider and budget for ongoing operational costs, including hardware maintenance, replacement of old devices, expanded server capacity (to account for increasing data over time), routine system maintenance and bug fixes and data bundles. These costs are commonly omitted or inaccurately budgeted; Vital Wave and Digital Square have developed a total cost of ownership tool for digital health that can be used to inform more accurate budget estimates [43].

Another important aspect of the enabling environment is the health workforce. Country leaders and partners should proactively address the needs of the health workforce given their essential role as end users of digital and data systems. The MVP design and testing should engage users to ensure it aligns with their processes, meets their needs and adds value for them. Other studies have shown that designing a system to add value directly to the people entering the data (e.g. by saving time in their daily tasks, providing clinical decision support, or providing data they can use to improve decision-making) can motivate them to use the system [6, 9]. In addition, engaging users for feedback can identify challenges (e.g. the limited search and edit functionalities reported in Kenya and Tanzania) early.

Our study found that HCW motivation to use the system was closely linked to having sufficient time to do so. Streamlining data management processes and aligning paper and digital tools can improve the efficiency of data entry. However, in an emergency response or campaign, country leaders or partners should mobilize additional staff to support short-term and/or high-volume data surges (e.g. for immunization or mass drug campaigns, new vaccine rollout or contact tracing) to reduce data backlogs. Investing in human resource information systems can help track, manage and plan for health workforce needs, including workforce shortages.

In DRC and Tanzania, HCWs also reported reduced motivation for data entry when they did not receive their salaries or compensation. Ministries of health should ensure timely payment of HCW salaries. One way to support this is by using digital financial services for direct payments to HCWs, which has been shown to increase efficiency and transparency for health systems and improve satisfaction for HCWs [44]. Donors and implementing partners could support the scale-up of such systems.

Finally, HCWs should be supported in their ongoing use of the digital and data systems. This may include refresher training, particularly if there are new iterations of the digital system or paper-based tools, or developing and introducing a system troubleshooting guide for end users to help them identify and address factors contributing to system slowdowns, freezes or crashes. Digital Square developed a DHIS2 Tracker troubleshooting guide that can be adapted for other contexts, health areas or systems [45, 46]. Additionally, information technology staff should be available to escalate any support requests users cannot address themselves. District or regional health managers can provide supportive supervision and timely feedback or lead data reviews, which other studies have shown can motivate health workers [6, 47].

### Limitations

This study was based on a non-representative sample of countries and subnational sites. The four study countries were purposefully selected by the study team based on USAID investments in COVID-19 vaccine digital and data systems, therefore the results may not be generalizable to all countries implementing similar systems. Within each country, subnational sites for primary data collection were purposefully selected in collaboration with each country’s MOH and were not designed to be nationally representative. In DRC, the study sites were limited to urban areas and the sample size was small (six sites). In Senegal, the final sample size (six sites) was smaller than initially designed due to the nationwide HCW strike during the time of data collection. Slightly different data collection tools and methods were used across the four countries, but the type of information captured was similar.

Although the study sample was not designed to be representative and methods varied by country, many common root causes were identified across contexts, and we believe the lessons from this study can inform other countries implementing large-scale data collection efforts. Given the scope of the research for this root cause analysis, these findings were not compared with digital implementations for COVID-19 in other countries. Additionally, we did not perform a cost analysis for the digital options chosen in each country compared to the previous manual paper-based systems.

## CONCLUSION

Root causes related to technology (system slowdowns and limited system functionality), infrastructure (insufficient devices and lack of reliable internet and data bundles), processes (incongruence between paper and digital tools, separate data collection and entry, lack of integration with routine immunization and lack of SOPs) and people (staff shortages, large workloads and non-payment of staff) contributed to observed COVID-19 data backlogs across DRC, Kenya, Senegal and Tanzania. Based on these findings, the study team identified three overarching recommendations for country leaders to inform large-scale data collection efforts using digital systems. First, use a country-led, coordinated approach to design and introduce digital and data systems, with planned iteration over time. Second, start with an MVP based on the critical data elements to inform decision-making and design the system based on the existing ecosystem. Third, proactively address the needs of the health workforce given their essential role as end users of digital and data systems. These lessons from the COVID-19 pandemic response can help inform broader health system strengthening efforts to improve effectiveness, resilience and pandemic preparedness of country health systems.
